# Modifiable factors associated with postoperative atrial fibrillation in older patients with hip fracture in an orthogeriatric care pathway: a nested case–control study

**DOI:** 10.1186/s12877-022-03556-9

**Published:** 2022-11-09

**Authors:** Pierre Gay, Bastien Genet, Audrey Rouet, Rana Alkouri, Judith Cohen-Bittan, Jacques Boddaert, Lorène Zerah

**Affiliations:** 1 Assistance Publique – Hôpitaux de Paris, Hôpital Pitié Salpêtrière, Département de gériatrie, Paris, France; 2 Assistance Publique – Hôpitaux de Paris, Hôpital Pitié Salpêtrière, Département de biochimie, Paris, France; 3 Sorbonne Université, INSERM, Centre d’Immunologie et des Maladies Infectieuses (Cimi), Paris, France; 4 Sorbonne Université, INSERM, Institut Pierre Louis d’Épidémiologie et de Santé Publique, Paris, France

**Keywords:** Postoperative atrial fibrillation, Older patients, Hip fracture, Orthogeriatric

## Abstract

**Background:**

Few data are available regarding post-operative atrial fibrillation (POAF) in non-cardiothoracic surgery, particularly orthopedic surgery. Hence, given the frequent incidence of POAF after surgery and its marked impact, we need to identify modifiable factors associated with POAF after hip fracture surgery in older patients.

**Methods:**

We conducted a nested case–control study in the unit for perioperative geriatric care of an academic hospital in Paris from July 1, 2009 to December 31, 2019, enrolling all consecutive patients aged ≥ 70 years with hip fracture surgery and no history of permanent AF before admission (retrospective analysis of prospectively collected data). Patients with and without POAF were matched 1:5 on 5 baseline characteristics (age, hypertension, diabetes, coronary artery disease, cardiac failure).

**Results:**

Of the 757 patients included, 384 were matched, and 64 had POAF. The incidence of POAF was 8.5%. The mean age was 86 ± 6 years, 298 (78%) patients were female, and the median Charlson Comorbidity Index was 6 (interquartile range 4–8). The median time from surgery to the occurrence of POAF was 2 days (1–4). On multivariable conditional logistic regression analysis (matched cohort), the modifiable factors present at admission associated with POAF were time to surgery > 48 h (odds ratio [OR] = 1.66, 95% confidence interval [1.01–2.81]) and > 2 units of packed red blood cells (OR = 3.94, [1.50–10.03]).

**Conclusions:**

This study provides new information about POAF in older patients with hip fracture surgery, a surgical emergency whose complexity requires multidisciplinary care.

**Supplementary Information:**

The online version contains supplementary material available at 10.1186/s12877-022-03556-9.

## Background

Hip fracture, with the total number of cases increasing in line with the ageing population, is a major public health concern associated with poor vital and functional outcomes [1–3]. In parallel, atrial fibrillation (AF), the most common sustained cardiac arrhythmia in adults, is particularly common in older patients [4], reaching 10% in patients over 80 years of age [5]. An increase in the number of surgical populations among older people over the last decade has increased the overall prevalence of postoperative AF (POAF), defined as new-onset AF in the immediate postoperative period: 20% to 50% of patients after cardiac surgery and 10% to 30% after non-cardiac thoracic surgery [4].

Postoperative AF, whose usual peak of incidence is between day 2 and 4 postoperatively [6], is associated with numerous complications such as cardioembolic stroke, acute coronary syndrome, and acute heart failure, with an impact on in-hospital mortality, length of hospital stay and medical health costs [4, 7–9]. Several studies, largely focused on POAF after cardiothoracic surgery, identified factors associated with POAF including baseline characteristics (age, male sex, hypertension, chronic heart failure, ischemic heart disease, chronic kidney disease), and perioperative and postoperative complications (hypovolemia, electrolyte disorders, hypoxemia) [4, 6, 9–12]. However, few data are available regarding POAF after non-cardiothoracic surgery, particularly emergent orthopedic surgery [4, 13].

Hence, given the frequent incidence of POAF after surgery and its marked impact, we need to identify factors, particularly modifiable factors, associated with POAF after orthopedic surgery. We previously demonstrated that optimizing patient care by improving the management of peri-operative factors and thus decreasing postoperative complications could reduce by a maximum of one quarter the 6-month mortality rate after hip fracture [14]. Identifying the modifiable factors associated with the occurrence of POAF in a population with multimorbidity and polypharmacy would be an essential step that could indicate the future directions for care improvement.

Our primary objective was to identify the modifiable factors associated with the occurrence of POAF in older patients with hip fracture surgery in a dedicated orthogeriatric pathway.

## Material and methods

The database was declared to the French National Commission on Computing and Liberty (CNIL) of the Assistance Publique-Hôpitaux de Paris (APHP) for this study (no. 20190822165316). This report follows the STROBE recommendations (Additional file 1) [15].

### Study design, study setting and eligibility criteria

A nested case–control study was conducted in the unit for perioperative geriatric care of the Pitié Salpêtriere hospital, a university hospital of the greater Paris University Hospitals Group (Assistance Publique Hôpitaux de Paris, AP-HP). The unit for perioperative geriatric care is part of a dedicated orthogeriatric care pathway including coordination between the department of emergency medicine and surgery, department of anaesthesiology and critical care, department of orthopaedic surgery and department of rehabilitation. This dedicated orthogeriatric care pathway is defined as 1) an early alert from the emergency department (ED), 2) considering hip fracture as requiring surgery as soon as feasible (i.e., 24 h/day), 3) rapid transfer to the unit for perioperative geriatric care after surgery, and 4) rapid transfer of stable patients to a dedicated rehabilitation unit [16, 17].

The management strategy in this unit for perioperative geriatric care, previously described [16], focused on early mobilization with the aim of chair-sitting and walking within 24 and 48 h after arrival, respectively; pain management; the provision of air-filled mattresses for patients with pressure sores or at high risk of pressure sores; swallowing disorders detected using a systematic medical survey; detection of stool impaction and urinary retention using bedside ultrasonography; correction of anemia with transfusion of packed red blood cells (haemoglobin level threshold ≤ 10 g/dl from 2009 to 2011 and haemoglobin level threshold ≤ 8 g /dl or symptoms since January 2012) [18]; and detection of delirium, AF and malnutrition. To screen for POAF, electrocardiography was systematically performed on admission to the unit for perioperative geriatric care. Then, electrocardiography was performed in case of tachycardia or irregular rhythm on clinical examination performed at least twice a day or hypotension, dyspnea, malaise, syncope, chest pain, delirium, anemia (haemoglobin level ≤ 8 g/dl) or electrolyte imbalance.

From July 1, 2009 to December 31, 2019, all consecutive patients with hip fracture admitted to the unit for perioperative geriatric care were evaluated for eligibility. Patients were included if they were ≥ 70 years old and their primary presentation was hip fracture (first hospitalization after surgery in the unit for perioperative geriatric care). On average, 150 to 200 patients aged ≥ 70 years old with hip fracture undergo hip surgery annually at Pitié Salpêtriere hospital, 75% of whom will be hospitalized in our unit for perioperative geriatric care. We excluded patients with multiple or metastatic or periprosthetic fractures, a history of permanent AF before admission in the unit for perioperative geriatric care, post-operative supraventricular arrhythmia other than AF; and missing data (missing anesthesia records, missing data from the ED, no electrocardiogram before admission in the unit for perioperative geriatric care). Patients were followed until death or the end of hospitalization in the unit for perioperative geriatric care. Some patients had been included in previous studies [14, 16–25].

### Outcomes

Our main outcome measure was the occurrence of POAF defined as new-onset AF in the immediate period after surgery (until the end of hospitalization in the unit for perioperative geriatric care). The occurrence of POAF was retrospectively adjudicated by 2 geriatricians (AR, PG) who independently reviewed medical charts (kappa = 0.948, 95% confidence interval [CI] 0.87–0.99). In case of disagreement, consensus was reached with a third independent senior expert (JB).

### Data collection methods and variables

Since the opening of the unit for perioperative geriatric care in 2009, we have created a dedicated research database that is prospectively implemented by 3 senior geriatricians (JB, JCB, LZ), experts in orthogeriatrics, and that integrates all the data from the orthogeriatric care pathway for each patient.

The following variables were collected prospectively by interviewing patients, their family members or their physicians and pharmacists during the hospital stay and were defined as baseline characteristics before hip fracture: age, sex, home or nursing home living conditions, walking ability, previous medical history including cardiovascular and neurologic diseases, chronic medications, and type of fracture (radiological definition by an orthopedic surgeon).

Co-morbidity severity was assessed with the Charlson Comorbidity Index [26] because the index, among a number of other comorbidity indexes, has been found to predict mortality in this population [20]. Functional status was evaluated with an activities of daily living scale (the Katz ADL index) [27]. Repeated falls was defined as 2 or more falls in the previous year, chronic renal failure as Cockcroft creatinine clearance < 30 ml.min^−1^, hypokalemia as potassium level < 3.5 mmol/L and anemia as haemoglobin level < 12 g.dL^−1^ for women and 13 g.dL^−1^ for men.

During the perioperative period, we prospectively recorded the surgical treatment, the delay and duration of surgery, the anesthetic drugs used and all drugs and transfusions administered from the ED to the unit for perioperative geriatric care. Cardiovascular drugs taken at baseline and their suspension were recorded retrospectively.

After surgery, delays to first sitting and first walking, destination (home or rehabilitation) at discharge from the unit for perioperative geriatric care and length of stay in acute care were recorded. All postoperative complications during the acute care period were prospectively recorded.

### Statistical analysis

The study is based on data for all available patients during the study period, and thus no a priori power calculation was conducted. Data are presented as mean ± SD or median (interquartile range) for continuous variables and number (percentage) for categorical variables. Comparison of quantitative variables involved unpaired Student *t* test or Mann–Whitney test depending on the normal distribution of data. Normality was assessed by graphical representation of the data distribution. Comparison of categorical variables involved chi-squared or Fisher’s exact test, as appropriate.

Patients with (cases) and without POAF (controls) were matched 1:5 on 5 baseline characteristics (age, hypertension, diabetes, coronary artery disease, cardiac failure). We selected those 5 factors from a literature review [4] and deliberations of a panel of 9 independent experts. This panel included 3 geriatricians, 4 cardiologists and 2 anesthesiologists who were all blinded to the research question at this time and to the other experts’ answers. We asked each expert the following standardized question: “According to you, what are the 9–10 main baseline predisposing factors of POAF among patients of 70 and more undergoing hip fracture surgery?” The 4 most frequently given answers were age (89%), diabetes (89%), hypertension (89%) and coronary artery disease (78%) (Additional file 2). Then, 4 other factors were mentioned with equal frequency: history of paroxysmal AF, chronic heart failure, chronic kidney disease, and valvular disease. We chose to keep chronic heart failure because it was most homogenously proposed by the 3 represented medical specialties and was previously described as a strong predisposing factor in this specific context [11, 28, 29]. This a priori and pragmatic method allows for improving the external validity and efficiency of the results and selecting a restricted number of baseline factors associated with POAF, in order to limit the number of patients excluded from the matching [30].

Then, using the matched dataset, we performed a conditional logistic regression analysis to assess independent modifiable variables present at admission in the unit for perioperative geriatric care that were associated with POAF; adjusted odds ratios (ORs) and their 95% CIs were calculated [30]. To avoid overestimation, a conservative approach was used [31, 32]: all variables with *P* < 0.20 on univariate analyses and all clinically relevant variables were included.

Finally, we performed a sensitivity analysis excluding patients with pre-existing AF before surgery (another definition of POAF).

Statistical analyses were performed with R v 1.4.1717 (package Matchlt). All p-values were two-tailed and *p* < 0.05 was considered statistically significant.

## Results

### Patient characteristics at baseline

**Fig. 1:**
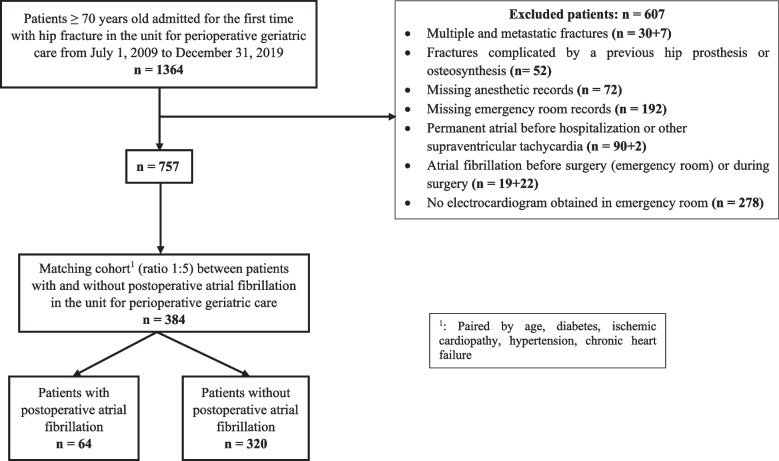
Flow chart of participants in the study

We included 757 patients, and 384 were matched; 64 had POAF (Fig. 1). Excluded patients (*n* = 607, 45%) had similar characteristics as patients included with one exception: fewer patients excluded had haemoglobin level < 10 g.dL^−1^ at admission in the unit for perioperative geriatric care (68% vs 74%, *p* = 0.03) (Additional file 3).

Baseline characteristics are reported in Table 1 (matched cohort). The mean age was 86 ± 6 years; 298 (78%) patients were female; the median Charlson Comorbidity Index was 6 (IQR 4–8); and 17% were living in a nursing home before the hip fracture.

**Table 1:** Demographic data and baseline characteristics of older patients with and without postoperative atrial fibrillation (POAF) (matched population)

	All patients *N* = 384	Without POAF *N* = 320	With POAF *N* = 64	*P* value
Age (years) ^a^	86 ± 6	86 ± 6	86 ± 6	0.99
< 85	141 (37)	116 (36)	25 (39)	0.67
85 to 90	145 (38)	122 (38)	23 (36)	0.74
> 90	98 (26)	82 (26)	16 (25)	0.92
Male sex	86 (22)	71 (22)	15 (23)	0.83
Medical history				
CCI	6 (4–8)	6 (4–8)	7 (4–7)	0.71
Paroxysmal atrial fibrillation	77 (20)	53 (17)	24 (38)	< 0.001*
Hypertension ^a^	264 (69)	220 (69)	44 (69)	0.99
Diabetes ^a^	24 (6)	20 (6)	4 (6)	0.99
Coronary artery disease ^a^	66 (17)	55 (17)	11 (17)	0.99
Cardiac failure ^a^	48 (13)	40 (13)	8 (13)	0.99
Valvular heart disease	30 (8)	22 (7)	8 (13)	0.13
Dementia	147 (38)	128 (40)	19 (30)	0.22
COPD	30 (8)	24 (8)	6 (9)	0.61
Chronic renal failure	35 (9)	29 (9)	6 (9)	0.99
Autonomy before surgery				
ADL	5.5 (4–6)	5.5 (3.5–6)	5.5 (5–6)	0.31
iADL	2 (0–4)	2 (0–4)	2 (1–3)	0.94
Living in institution	65 (17)	64 (20)	1 (2)	< 0.001*
Walking before surgery	373 (97)	309 (97)	64 (100)	0.13
Walking with assistance	227 (59)	186 (58)	41 (64)	0.38
Fracture				
Intertrochanteric fracture	206 (54)	169 (53)	37 (58)	0.48
Femoral neck fracture	156 (41)	133 (42)	23 (36)	0.39
Surgery				
Time to surgery (hr)	26 (20–47)	26 (19–46)	33 (20–54)	0.11
Time to surgery > 48 h	85 (22)	62 (19)	23 (36)	0.004*
Duration of surgery (min)	143 ± 48	141 ± 43	151 ± 68	0.13
*Missing values*	*4 (1)*	*4 (1)*	*0 (0)*	*-*
Type of surgery				
Gamma nail	196 (51)	162 (51)	34 (53)	0.79
Dynamic hip screw	22 (6)	17 (5)	5 (8)	0.39
Unipolar prosthesis	138 (36)	117 (37)	21 (33)	0.52
Bipolar prosthesis	12 (3)	10 (3)	2 (3)	0.99
*Missing values*	*4 (1)*	*4 (1)*	*0 (0)*	*-*
Laboratory values				
Haemoglobin level at admission g.dL^−1^	9.0 [8.0–9.9]	9.0 [7.9–9.9]	9.0 [8.2–10.0]	0.48
Haemoglobin level < 10 g.dL^−1^ at admission	292 (76)	245 (77)	47 (73)	0.56
*Missing values*	*1 (0)*	*1 (0)*	*0 (0)*	*-*
Kalemia < 3.5 mmol/L at admission	42 (11)	34 (11)	8 (13)	0.66
*Missing values*	*32 (8)*	*27 (8)*	*5 (8)*	*-*

Data are mean ± SD, median (interquartile range), or number (percentage). Missing values are specified only if present

Abbreviations: *CCI* Charlson comorbidity, *COPD* chronic obstructive pulmonary disease, chronic renal failure creatinine clearance < 30 ml/min, *ADL* Activities of daily living, *iADL* index of activities of daily living

^a^Matching on these 5 characteristics

The incidence of POAF was 8.5% in our cohort (Fig. 1), with a median time between hospitalization in our unit and the occurrence of POAF of 2 days (1–4). On univariate analysis (matched cohort), patients with and without POAF did not differ in comorbidities (except for history of paroxysmal AF), autonomy, type of hip fracture or surgery (Table 1). The median time to surgery was 26 h (20–47), and for more patients with than without POAF, the time to surgery was longer than 48 h: 23 (36%) versus 62 (19%) (*p* = 0.004). The groups did not differ in laboratory values at admission in the unit for perioperative geriatric care (haemoglobin and potassium levels) (Table 1).

### Perioperative therapeutics

On univariate analysis (matched cohort), patients with and without POAF did not differ in use of anesthetic drugs or techniques (Table 2). Patients with POAF more frequently received more than 2 units of packed red blood cells in the emergency room or recovery room than patients without POAF: 8 (13%) versus 9 (3%) (*p* = 0.003) (Table 2). The groups did not differ in cardiovascular drug treatment at baseline, with the exception of flecainide, used more at baseline in the POAF than non-POAF group (Additional file 4).

**Table 2:** Management, complications and in-hospital outcomes of older patients with and without postoperative atrial fibrillation (POAF) (matched population)

	All patients *N* = 384	Without POAF *N* = 320	With POAF *N* = 64	*P* value
Perioperative therapeutics				
General anesthesia	342 (89)	285 (89)	57 (89)	0.89
*Missing values*	*31 (8)*	*26 (8)*	*5 (8)*	*-*
Femoral block	154 (40)	12 (4)	31 (48)	0.17
*Missing values*	*37 (10)*	*32 (10)*	*5 (8)*	*-*
Morphinics	338 (88)	280 (88)	58 (91)	0.73
*Missing values*	*38 (10)*	*33 (10)*	*5 (8)*	*-*
Hypnotics	333 (87)	275 (86)	58 (91)	0.36
*Missing values*	*38 (10)*	*33 (10)*	*5 (8)*	*-*
Fluranes	200 (52)	167 (52)	33 (52)	0.42
*Missing values*	*80 (21)*	*70 (22)*	*10 (16)*	*-*
Catecholamines	301 (78)	252 (79)	49 (77)	0.32
*Missing values*	*39 (10)*	*34 (11)*	*5 (8)*	*-*
Others				
Prostigmin	14 (4)	13 (4)	1 (2)	0.48
*Missing values*	*40 (10)*	*35 (11)*	*5 (8)*	*-*
Atropine	25 (7)	21 (7)	4 (6)	0.99
*Missing values*	*39 (10)*	*34 (11)*	*5 (8)*	*-*
Transfusion in ER or RR	111 (29)	88 (28)	23 (36)	0.16
> 2 units of packed RBCs	17 (4)	9 (3)	8 (13)	0.003*
*Missing values*	*3 (1)*	*3 (1)*	*0 (0)*	*-*
In-hospital complications				
Infection	68 (18)	51 (16)	17 (27)	0.04*
Acute heart failure	36 (9)	23 (7)	13 (20)	0.001*
Acute coronary syndrome	27 (7)	20 (6)	7 (11)	0.18
Thromboembolic disease	27 (7)	23 (7)	4 (6)	0.99
Stroke	4 (1)	0 (0)	4 (6)	< 0.001*
Discharge				
In-hospital mortality	7 (2)	5 (2)	2 (3)	0.33
Back home at discharge	229 (60)	190 (59)	39 (61)	0.82
Length of stay (days)	11 ± 6.1	11 ± 5.5	13 ± 8.5	0.007*

Data are mean ± SD, median (interquartile range), or number (percentage). Missing values are specified only if present

Abbreviations: *ER* Emergency room, *RR* Recovery room, *RBC* Red blood cell

### Main outcome

On multivariable conditional logistic regression analysis (matched cohort; all variables detailed in Table 3), the modifiable factors associated with POAF, present at admission in the unit for perioperative geriatric care, were time to surgery > 48 h (OR = 1.66 [1.01–2.81]) and transfusion of > 2 units of packed red blood cells in the emergency room or recovery room (OR = 3.94 [1.50–10.03])*.*

**Table 3:** Logistic regression analysis of factors associated with occurrence of postoperative atrial fibrillation (POAF) (matched population)

	**Univariate analysis OR (95% CI)**	***P* value**	**Multivariableanalysis OR (95% CI)**	***P* value**
**Baseline**				
CCI (for 1-point increase)	0.94 (0.86–1.02)	0.15	0.96 (0.85–1.09)	0.54
Male sex	1.08 (0.74–1.59)	0.70	1.19 (0.71–2.01)	0.52
History of paroxysmal atrial fibrillation	2.45 (1.70–3.54)	< 0.001*	2.94 (1.75–4.92)	0.001*
Time to surgery > 48 h	2.36 (1.62–3.43)	< 0.001*	1.66 (1.01–2.81)	0.05*
Duration of surgery (min)	1.01 (1.00–1.02)	0.03*	0.99 (0.99–1.01)	0.18
Hypokalemia	1.16 (0.68–1.98)	0.59	1.68 (0.84–3.35)	0.14
**Therapeutics**				
Femoral block	1.54 (1.10–2.15)	0.01*	0.86 (0.56–1.33)	0.50
> 2 units of packed RBCs in ER or RR	4.88 (2.28–10.4)	< 0.001*	3.94 (1.50–10.3)	0.005*

*N* 321 (cases: 54, controls: 267), Concordance = 0.69

Hypokalemia = potassium level < 3.5 mmol.l^−1^ at admission in unit for perioperative geriatric care

Abbreviations: *OR* Odds ratio, *CI* Confidence interval, *CCI* Charlson comorbidity index, *RBC* Red blood cell, *ER* Emergency room, *RR* Recovery room

Sensitivity analyses excluding patients with pre-existing AF before surgery revealed that modifiable factors associated with POAF, present at admission, were time to surgery > 48 h (OR = 2.79 [1.33–5.86]) and hypokalemia (OR = 2.50 [1.06–5.87]) but not transfusion of > 2 units of packed red blood cells in the emergency room or recovery room (OR = 3.25 [0.99–10.6]) (Additional file 5).

### Post-operative complications

On univariate analysis (matched cohort), stroke, acute heart failure and infection were more frequent in patients with than without POAF (Table 2). In 5 patients with acute heart failure (AHF), the AHF occurred before the discovery of POAF. Also, in 3 patients with acute coronary syndrome (ACS), the ACS occurred before the discovery of POAF.

## Discussion

This study aimed to identify modifiable factors associated with POAF after hip fracture surgery in a geriatric population receiving treatment in a dedicated orthogeriatric care pathway. The incidence of POAF was 8.5% in our cohort. The occurrence of POAF was associated with time to surgery greater than 48 h, and more than 2 units of packed red blood cells before hospitalization in the unit for perioperative geriatric care.

The reported incidence of POAF after non-thoracic non-cardiac surgery varies widely, from 0.5% to 15%, which reflects the heterogeneity in definitions (inclusion or not of patients with a history of AF), methods used to identify POAF, study populations (geriatric or not) and settings (type of surgeries) [4, 6, 13]. We chose to include in this cohort patients with a history of paroxysmal AF, as for previous cohorts of patients with hip fracture.[10, 11]. In studies by Leibowitz et al.[11] (*n* = 410, mean age 80 ± 7.8 years) and Rostagno et al. [10] (*n* = 2922, mean age 83.7 ± 8.2 years), the incidence of newly diagnosed AF among older comorbid patients undergoing hip fracture surgery was 3.7% and 3.6%, respectively. Because the definition of POAF and the populations were similar to our cohort [10, 11], the higher incidence we found in our cohort may have been related to differences in monitoring and screening POAF in this highly specialized environment [14, 16].

We found delayed surgery (usually due to preoperative medical assessment, anticoagulant interruption and access to the operating room [14, 17]) associated with the occurrence of POAF, which agrees with the study of Rostagno et al., finding a time to surgery less than 48 h for 80% of patients in the control group versus 49% in the AF group (*p* = 0.0001) [10]. In this case, pain, anemia, electrolyte imbalance, therapeutics management and/or infection may be involved [10]. Surgery for a hip fracture should be considered an emergency. A meta-analysis including 191 873 patients found that early hip fracture surgery (cut-off between 24 and 48 h) was associated with significantly reduced risk of death (OR = 0.74 [0.67–0.81]) [33]. Of note, reducing the time to surgery is not included in the European Society of Cardiology recommendations to prevent POAF [4], but our results support the optimization of the perioperative way of care allowing for a short surgical delay.

We also found the occurrence of POAF associated with more than 2 units of packed of red blood cells before hospitalization in the unit for perioperative geriatric care, which may reflect complications of anemia or the transfusion itself [34, 35]. This result was not confirmed by a sensitivity analysis excluding patients with pre-existing AF prior surgery, possibly due to lack of power. Perioperative anemia is a known factor associated with POAF. Anemia may lead to relative ischemia of atrial cells and myocardial conduction tissue, thus altering the cell electric properties and leading to arrhythmias [12, 36]. In addition, anemia, especially acute anemia, produces an intense adrenergic activation that may trigger POAF in predisposed patients [12, 36]. However, in cardiothoracic surgery, there is evidence that packed red blood cell transfusion increases the incidence of POAF [37]. We previously found in our unit that a restrictive transfusion strategy was associated with fewer in-hospital cardiovascular complications (including AF, ACS, AHF and stroke) with no significant difference in long-term mortality in older patients with hip fracture [18]. Data are limited on the impact of restrictive versus liberal transfusion strategy on cardiac injury in older patients undergoing hip fracture surgery.[38], with no difference between transfusion regimens in the FOCUS study including cardiovascular outcomes [34]. In the ongoing RESULT-Hip trial [39], patients ≥ 60 years undergoing surgery for hip fracture who become anaemic (haemoglobin level < 9 g/dl) are randomly assigned to a liberal transfusion strategy (target haemoglobin level 9–11 g/dl) or restrictive transfusion strategy (target haemoglobin level 7.5–9 g/dl) during their hospitalization. The primary outcome is death or major adverse cardiovascular events, including new arrhythmia, within 30 days of surgery (starting date 2022). This trial should provide relevant answers in a few years.

We found an association between hypokalemia and POAF only in our sensitivity analysis [36], which suggests that POAF may be triggered by different factors depending on the underlying cardiopathy and cardiac vulnerability. In addition, we collected the serum potassium level only on the first day of hospitalization, which could explain why we did not find an association of hypokalemia with POAF occurring later during the hospital stay. We found no association with cardiovascular drug use [4, 6], possibly owing to lack of power.

Our study has several limitations. First, it was an observational study, and causality cannot be demonstrated. Second, it was a retrospective study, and bias due to other procedural changes, which may have influenced the occurrence of POAF during the same time cannot be excluded. Third, many patients had to be excluded because of missing preoperative electrocardiogram results (including missing anesthesia or emergency records) to fully meet the definition (exclusion of permanent AF). However, these patients had the same characteristics as included patients with the exception that they had less aenemia before surgery (potential factor associated with POAF). Fourth, we were unable to collect the minimum haemoglobin value for all patients before their admission in our unit. Transfusion in the emergency room or recovery room, especially > 2 units of packed RBCs, may be a proxy for severe anaemia during this perioperative period. Fifth, patients were not continuously monitored, which may have resulted in an under-diagnosis of the incidence of paroxysmal POAF and misclassification bias. Sixth, because our study was conducted in a highly specialized environment that is associated with reduced mortality and postoperative complications as compared with patients admitted to an orthopaedic department [14, 16], our results may not be extrapolated to other wards.

This study may have significant implications for the care of perioperative patients. The complexity of POAF, in particular in a population with multimorbidity and polypharmacy, requires a multifaceted, holistic, and multidisciplinary approach to the management of AF patients, with active involvement in partnership with geriatricians, surgeons, anesthetists and emergency physicians in an optimized perioperative way of care [4].

## Conclusions

Among older patients hospitalized in a dedicated orthogeriatric care pathway for hip fracture surgery, the occurrence of POAF was associated with 2 modifiable factors: time to surgery greater than 48 h and more than 2 units of packed of red blood cells before the hospitalization in the unit for perioperative geriatric care. Further studies are needed to demonstrate a possible causal link between these factors and POAF.

## Supplementary Information

**Additional file 1.** STROBE Statement—Checklist of items that should be included in reports of *cohort studies*.

**Additional file 2.** Answers of 9 atrial fibrillation experts to the question: “According to you, what are the 9 or 10 main baseline predisposing factors of POAF among patients of 70 and more undergoing HF surgery?”

**Additional file 3.** Comparison between included and excluded patients in the perioperative geriatric unit.

**Additional file 4.** Cardiovascular drugs before surgery at baseline in patients hospitalized in the perioperative geriatric unit with and without post-operative atrial fibrillation (POAF).

**Additional file 5.** Sensitivity analysis: exclusion of patient with pre-existing atrial fibrillation prior to surgery.

## Data Availability

The datasets analysed during the current study are available from author Pr Jacques Boddaert on reasonable request (jacques.boddaert@aphp.fr).

## Abbreviations

AF: Atrial fibrillation
POAF: Postoperative atrial fibrillation
ED: Emergency department
CNIL: Commission nationale de l’informatique et des libertés
APHP: Assistance publique–hopitaux de Paris
