# Supplementary material for: Modifiable factors associated with postoperative atrial fibrillation in older patients with hip fracture in an orthogeriatric care pathway: a nested case–control study
Source: BMC Geriatr. 2022 Nov 9;22:845. doi: 10.1186/s12877-022-03556-9 (PMC9644640; doi:10.1186/s12877-022-03556-9)
Supplement: Supplementary file 2 — Additional file 2. Answers of 9 atrial fibrillation experts to the question: “According to you, what are the 9 or 10 main baseline predisposing factors of POAF among patients of 70 and more undergoing HF surgery?” [file 12877_2022_3556_MOESM2_ESM.docx]

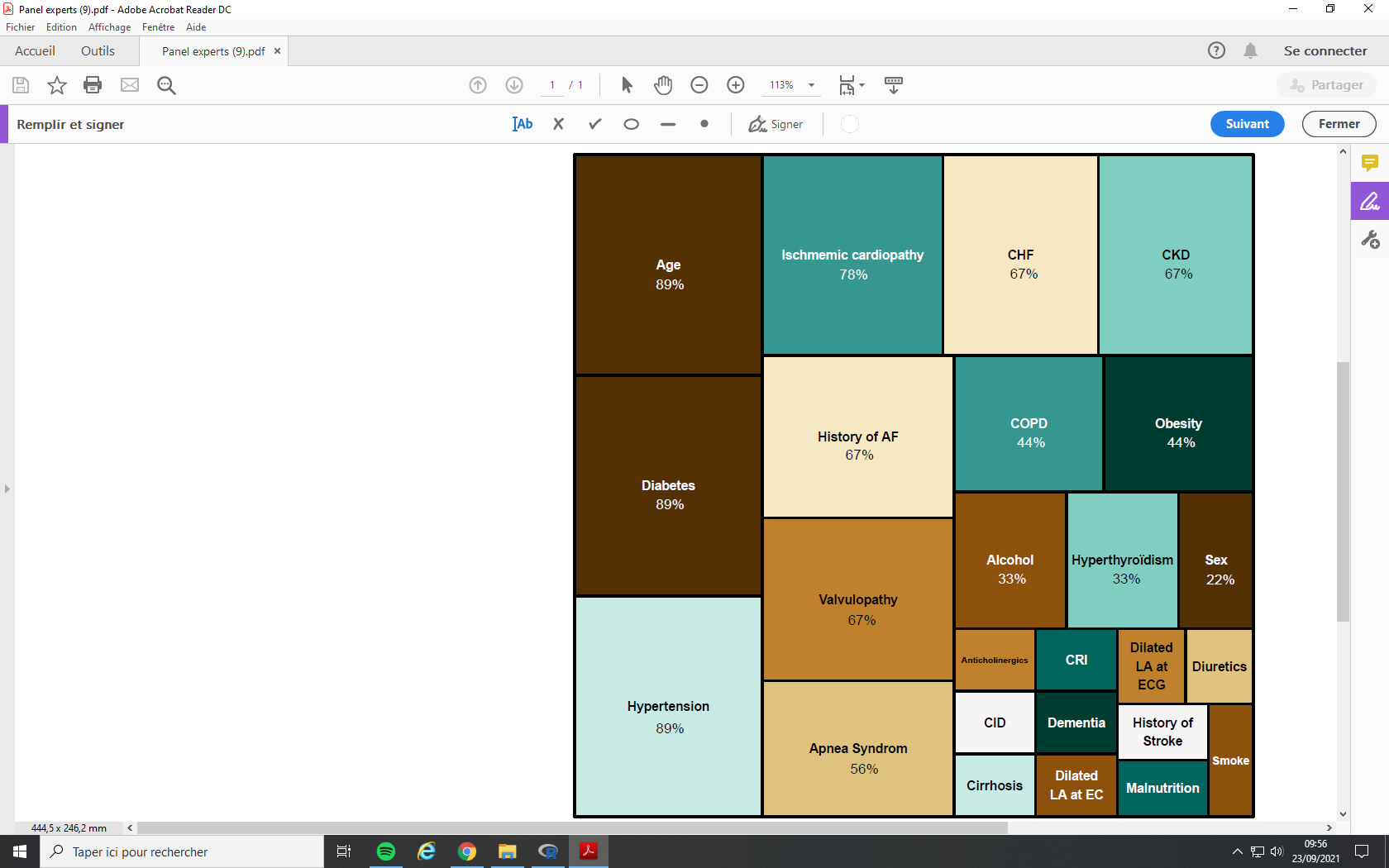
**Additional file 2: Answers of 9 atrial fibrillation experts to the question: “According to you, what are the 9 or 10 main baseline predisposing factors of POAF among patients of 70 and more undergoing HF surgery?”**

Note: When the percentage is not indicated, it is equal to 11%

Abbreviations: CKD = chronic kidney disease, AF = atrial fibrillation, CHF = chronic heart failure, COPD = chronic obstructive pulmonary disease, CID = chronic inflammatory disease, CRI = chronic respiratory insufficiency, LA = left atrium, ECG = electrocardiography, EC = echocardiography

3 Geriatricians (5 more frequent): age (100%), diabetes (100%), hypertension (100%), ischemic cardiopathy (100%), valvulopathy (100%)

2 Anesthesiologists (16 more frequent): ischemic cardiopathy (100%), history of AF (100%), CKD (100%), age (50%), hypertension (50%), diabetes (50%), CHF (50%), valvulopathy (50%), COPD (50%), alcohol (50%), anticholinergics (50%), history of stroke (50%), cirrhosis (50%), smoking (50%), malnutrition (50%), dementia (50%)

4: Cardiologists (8 more frequent): age (100%), diabetes (100%), hypertension (100%), history of AF (75%), CHF (75%), CKD (75%), apnea syndrome (75%), obesity (75%)

**5 items retained for the matching: age (89%), diabetes (89%), hypertension (89%), ischemic cardiopathy (78%), CHF (67%)**
