# Supplementary material for: Modifiable factors associated with postoperative atrial fibrillation in older patients with hip fracture in an orthogeriatric care pathway: a nested case–control study
Source: BMC Geriatr. 2022 Nov 9;22:845. doi: 10.1186/s12877-022-03556-9 (PMC9644640; doi:10.1186/s12877-022-03556-9)
Supplement: Supplementary file 3 — Additional file 3. Comparison between included and excluded patients in the perioperative geriatric unit. [file 12877_2022_3556_MOESM3_ESM.docx]

**Additional file 3: Comparison between included and excluded patients in the perioperative geriatric unit**

|  | **All patients**  **N = 1364** | **Included patients**  **N = 757** | **Excluded patients**  **N = 607** | **P Value** |
| --- | --- | --- | --- | --- |
| Age (years) | 86 ± 6 | 86 ± 6 | 87 ± 6 | 0.23 |
| > 85 years old | 802 (59) | 437 (58) | 365 (60) | 0.37 |
| Male sex | 330 (24) | 175 (23) | 155 (26) | 0.30 |
| Medical history |  |  |  |  |
| CCI | 7 (4-8) | 7 (4-8) | 7 (4-8) | 0.65 |
| Diabetes | 188 (14) | 97 (13) | 91 (15) | 0.25 |
| Hypertension | 918 (67) | 498 (66) | 420 (69) | 0.18 |
| Coronary artery disease | 242 (18) | 137 (18) | 135 (22) | 0.70 |
| Cardiac failure | 225 (16) | 115 (15) | 110 (18) | 0.15 |
| Stroke | 238 (17) | 125 (17) | 113 (19) | 0.31 |
| Valvular heart disease | 112 (8) | 59 (8) | 53 (9) | 0.53 |
| COPD | 107 (8) | 59 (8) | 48 (8) | 0.94 |
| Chronic renal failure | 146 (11) | 77 (10) | 69 (11) | 0.48 |
| Biologic factors |  |  |  |  |
| Haemoglobin < 10 g.dL^-1^ at admission | 974 (71) | 559 (74) | 415 (68) | 0.03* |
| Fracture |  |  |  |  |
| Intertrochanteric fracture | 674 (49) | 377 (50) | 297 (49) | 0.80 |
| Femoral neck fracture | 602 (44) | 337 (45) | 265 (44) | 0.80 |
| Surgery |  |  |  |  |
| Time to surgery (hr) | 27 (19-48) | 26 (19-46) | 28 (19-50) | 0.31 |
| Time to surgery > 48 hr | 319 (23) | 165 (22) | 154 (25) | 0.07 |
| Duration of surgery (min) | 140 ± 50 | 141 ± 52 | 139 ± 48 | 0.45 |
| Gamma nail | 660 (48) | 371 (49) | 289 (48) | 0.73 |
| Dynamic hip screw | 74 (5) | 41 (5) | 33 (5) | 0.96 |
| Unipolar prosthesis | 514 (38) | 287 (38) | 228 (38) | 0.97 |
| Bipolar prosthesis | 53 (4) | 27 (4) | 26 (4) | 0.48 |

Data are mean ± SD, median (interquartile range), or number (percentage).

Abbreviations: CCI = Charlson Comorbidity Index; COPD = chronic obstructive pulmonary disease; chronic renal failure = creatinine clearance <30 ml.min^-1^.
