# Supplementary material for: Modifiable factors associated with postoperative atrial fibrillation in older patients with hip fracture in an orthogeriatric care pathway: a nested case–control study
Source: BMC Geriatr. 2022 Nov 9;22:845. doi: 10.1186/s12877-022-03556-9 (PMC9644640; doi:10.1186/s12877-022-03556-9)
Supplement: Supplementary file 4 — Additional file 4. Cardiovascular drugs before surgery at baseline in patients hospitalized in the perioperative geriatric unit with and without post-operative atrial fibrillation (POAF). [file 12877_2022_3556_MOESM4_ESM.docx]

**Additional file 4: Cardiovascular drugs before surgery at baseline in patients hospitalized in the perioperative geriatric unit with and without post-operative atrial fibrillation (POAF)**

|  | **All patients**  **N = 384** | **Without POAF**  **N = 320** | **With POAF**  **N = 64** | **P value** |
| --- | --- | --- | --- | --- |
| **ACEi and ARBs** | 132 (34) | 109 (34) | 23 (36) | 0.77 |
| **Beta-blockers** | 93 (24) | 78 (24) | 15 (23) | 0.86 |
| **Statins** | 53 (14) | 69 (22) | 14 (22) | 0.96 |
| **Amiodarone** | 46 (12) | 35 (11) | 11 (17) | 0.16 |
| **Flecainide** | 9 (2) | 5 (2) | 4 (6) | 0.04* |
| **Propafenone** | 1 (0) | 0 (0) | 1 (2) | 0.99 |
| **Digoxin** | 4 (1) | 4 (1) | 0 (0) | 0.99 |
| **Antiplatelet** | 147 (38) | 125 (39) | 22 (34) | 0.70 |
| **Oral anticoagulant** | 50 (13) | 40 (13) | 10 (16) | 0.74 |

Data are number (percentage). Missing value are specified only if they were present

Abbreviations: ACEi = angiotensin converting enzyme inhibitor; ARBs = angiotensin-II receptor block
