# Supplementary material for: Modifiable factors associated with postoperative atrial fibrillation in older patients with hip fracture in an orthogeriatric care pathway: a nested case–control study
Source: BMC Geriatr. 2022 Nov 9;22:845. doi: 10.1186/s12877-022-03556-9 (PMC9644640; doi:10.1186/s12877-022-03556-9)
Supplement: Supplementary file 5 — Additional file 5. Sensitivity analysis: exclusion of patient with pre-existing atrial fibrillation prior to surgery. [file 12877_2022_3556_MOESM5_ESM.docx]

**Additional file 5: Sensitivity analysis: exclusion of patient with pre-existing atrial fibrillation prior to surgery**


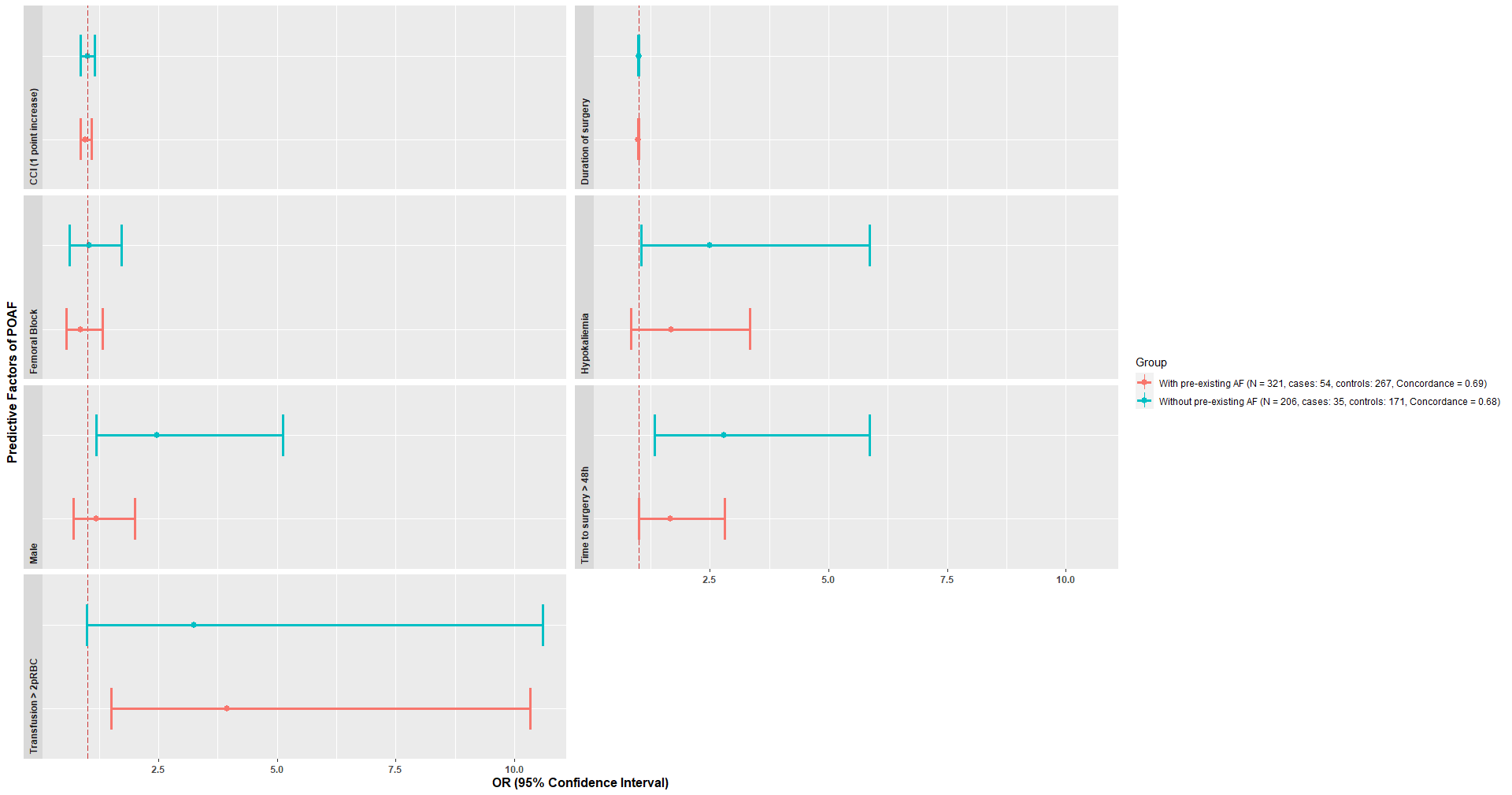
Abbreviations: CCI = Charlson Comorbidity Index; RBC = red blood cell, POAF postoperative atrial fibrillation, AF: atrial fibrillation

Hypokalemia = potassium level <3.5 mmol.l^-1^ at admission in unit for perioperative geriatric care
